# Simulated Annealing–Guided Geometric Descent-Optimized Frequency-Domain Compression-Based Acquisition Algorithm

**DOI:** 10.3390/s26010220

**Published:** 2025-12-29

**Authors:** Fangming Zhou, Wang Wang, Yin Xiao, Chen Zhou

**Affiliations:** 1 School of Aerospace Engineering, Geely University of China, Chengdu 641423, China; zhoufangming@guc.edu.cn (F.Z.); wangwang@guc.edu.cn (W.W.); 2 School of Earth and Space Science and Technology, Wuhan University, Wuhan 430072, China; chenzhou@whu.edu.cn

**Keywords:** GNSS signal acquisition, compressed acquisition, doppler frequency offset, high-dynamic environments, sparse recovery

## Abstract

Global Navigation Satellite System (GNSS) signal acquisition in high-dynamic environments faces significant challenges due to large Doppler frequency offsets and stringent computational constraints. This paper proposes a frequency-domain compressed acquisition algorithm that reformulates the conventional two-dimensional code-phase/Doppler search as a set of independent one-dimensional sparse recovery problems. Doppler uncertainty is modeled as sparsity in a discretized frequency dictionary, and a low-coherence measurement matrix is designed offline via projected gradient descent with a two-stage annealing strategy. The resulting matrix significantly reduces maximum coherence and supports reliable sparse recovery from a small number of compressed measurements. During online operation, the receiver forms compressed observations for all code phases through efficient matrix operations and recovers sparse Doppler spectra using lightweight orthogonal matching pursuit. Simulation results show that the proposed method achieves a several-fold reduction in computational cost compared with classical parallel code-phase search while maintaining high detection probability at low carrier-to-noise density ratios and under large Doppler offsets, providing an effective solution for resource-constrained GNSS receivers in high-dynamic scenarios.

## 1. Introduction

Global Navigation Satellite Systems (GNSS) have become increasingly prevalent in high-speed aircraft communication scenarios [1,2,3,4,5], driving the need for low-power, high-sensitivity receivers. In communication networks with constrained processing resources and extensive spatial coverage, GNSSs increasingly serve as the primary real-time positioning means; low-complexity, high-sensitivity acquisition algorithms can broaden feasible applications and improve overall system performance. However, in high-speed aircraft scenarios, large relative velocities and accelerations between aircraft and satellites produce substantial Doppler frequency offsets and higher-order terms, posing significant challenges for GNSS signal acquisition in high-dynamic environments. Here, high dynamics mainly manifest as large Doppler uncertainty (large Doppler offsets and potential Doppler-rate effects) that significantly enlarges the frequency search space and challenges real-time acquisition.

Doppler frequency offsets in high-mobility environments strongly affect communication system performance, particularly in GNSS precision positioning. To address Doppler compensation in high dynamic environments, Stirling-Gallacher et al. [6] and Spangenberg et al. [7] systematically analyzed acquisition algorithms combining fast Fourier transforms (FFTs) with digital matched filters and proposed the partial match filter-fast Fourier transform (PMF-FFT) algorithm. Unfortunately, this approach is computationally intensive and requires increased FFT points to mitigate frequency-offset leakage, further increasing resource consumption. To alleviate the heavy hardware demand of the PMF stage, Qi et al. [8] developed an improved scheme employing folded matched filters to accelerate computation. Li et al. [9] proposed a novel code-acquisition algorithm improving detection probability at constant algorithmic complexity for a given false-alarm probability. To estimate and remove Doppler shifts, Qin et al. [10] introduced an inertial navigation system-assisted acquisition scheme based on parallel code-phase search, reducing the frequency search space and improving acquisition efficiency. Le et al. [11] designed a carrier-acquisition algorithm based on delay autocorrelation that shortens acquisition time and computational cost while enabling rapid estimation of initial carrier Doppler frequency and its rate of change. Zhao et al. [12] proposed a fast acquisition method based on refined coherent averaging, combining FFT processing, coherent integration, and averaging correlation to shorten acquisition time for high-dynamic satellite signals and improve acquisition accuracy.

To further accelerate the search over Doppler frequency shifts, compressive folding of the input signal has been introduced into acquisition schemes. Kong [13] proposed superimposing subcarriers at different local frequency points, then performing quadrature down-conversion with the received signal for acquisition, shortening acquisition time. He et al. [14] incorporated compressive sensing into acquisition algorithms, using the Kronecker product of frequency indices and code phases as a transform matrix for compressed acquisition to reduce acquisition time. However, matrices generated by the Kronecker construction are extremely large, leading to high computational complexity in signal reconstruction. To address this, Zhou et al. [15] proposed a code–frequency dual-segment compression algorithm. Here, the input signal is first mapped and superposed onto subcarriers at different frequency points to mitigate large frequency offsets, then code-domain compressed acquisition is performed on the preprocessed frequency-domain signal to reduce resource consumption in high-dynamic environments. Nevertheless, this algorithm yields a relatively low detection probability for small Doppler offsets.

In parallel, Deng et al. [16] improved compressed sensing (CS)-based acquisition by constructing a singular value decomposition (SVD)-based Gaussian measurement matrix with enhanced mutual incoherence, achieving higher acquisition probability at low SNR (signal-to-noise ratio). Building on this, Zhang et al. [17] combined SVD-enhanced Gaussian matrices with partial matched filter–FFT (PMF–FFT) preprocessing, reaching near-conventional performance with far fewer operations.

In summary, prior studies on compressive sensing (CS)-based GNSS acquisition have mainly emphasized sensing-matrix design and the associated sparse-recovery algorithms. However, investigations tailored to high-dynamic conditions—characterized by large Doppler shifts and high Doppler rates—remain limited. To bridge this gap, we develop a frequency-domain compressed acquisition scheme that constructs a low-coherence measurement matrix by explicitly shaping its Gram structure and integrates the resulting deterministic operator into a frequency-domain CS workflow for rapid acquisition.

Compared with dual-domain compression methods that rely on multi-stage signal folding/mapping before compressed acquisition, and with recent singular value decomposition (SVD)-based CS schemes that modify random Gaussian matrices without directly optimizing the Gram structure of the acquisition dictionary, our approach directly targets the sensing operator itself. Specifically, we propose a simulated-annealing-guided geometric descent (SAGD) strategy that minimizes the maximum mutual coherence by steering the Gram matrix toward a more decorrelated (low-interference) structure. This provides a controllable mechanism for improving peak separability in greedy sparse recovery. By embedding the optimized deterministic matrix into the frequency-domain compressive acquisition pipeline, the receiver achieves robust acquisition under large Doppler uncertainty using fewer measurements and reduced search complexity, which is well suited to high-dynamic and resource-constrained scenarios.

The contributions of this paper are summarized below:

1. A frequency-domain compressed GNSS signal acquisition method reducing the dimensionality of the frequency search space by constructing sparse bases for the Doppler frequency offsets.
2. A SAGD-based measurement-matrix design method via Gram matrix shaping with a two-stage annealing schedule, which substantially reduces the maximum mutual coherence compared with common random/structured baselines.
3. A complete theoretical analysis framework for GNSS signal acquisition under compressive sensing, elucidating how compression ratio and matrix coherence jointly influence detection performance.

The remainder is organized as follows. Section 2 analyzes the impact of Doppler frequency offsets on acquisition performance in high-dynamic environments and introduces the frequency domain compressive sensing scheme. Section 3 describes measurement matrix optimization methods and the overall algorithm workflow in detail. Section 4 presents theoretical performance analysis. Section 5 provides simulation results and discussions, followed by concluding remarks.

## 2. Signal Model

### 2.1. Signal Model and Doppler Frequency Offset

In GNSS signal reception, the received intermediate-frequency (IF) signal can be modeled as

(1) $$r\left(t\right)=A\;d\left(t\right)\;c\left(t-\tau \right)\cos\;\left(2\pi (f_{\mathrm{IF}}+f_{d})t+\varphi \right)+n\left(t\right),$$

where *A* is the signal amplitude, d(t) is the navigation data, c(t) is the spreading code, $f_{\mathrm{IF}}$ is the intermediate frequency, $f_{d}$ is the Doppler frequency offset, τ is the code delay, φ is the initial carrier phase, and n(t) is additive white Gaussian noise [18].

After downconversion and low-pass filtering, the complex baseband signal is

(2) $$r_{\mathrm{bb}}\left(t\right)=A\;d\left(t\right)\;c\left(t-\tau \right)\;e^{j(2\pi f_{d}t+\varphi )}+n_{\mathrm{bb}}\left(t\right),$$

where $n_{\mathrm{bb}}\left(t\right)$ denotes complex baseband noise.

Neglecting navigation data bit transitions over the coherent integration interval and absorbing φ into the complex amplitude, the discrete-time baseband model becomes

(3) $$r\left[n\right]=A\;c\left[n-n_{\tau }\right]\;e^{j2\pi f_{d}nT_{s}}+w\left[n\right],$$

where $T_{s}$ is the sampling interval, $w\left[n\right]∼\mathrm{CN}\left(0,\sigma_{w}^{2}\right)$ is discrete-time noise, and $n_{\tau }≜\tau /T_{s}$ is the (possibly non-integer) code delay in samples.

### 2.2. Correlation Function Analysis

Given local replica hypotheses $\left({\hat{f}}_{d},{\hat{n}}_{\tau }\right)$, the coherent correlation statistic is

(4) $$R\left({\hat{f}}_{d},{\hat{n}}_{\tau }\right)=\sum_{n=0}^{N_{\mathrm{sample}}-1}r\left[n\right]\;c^{*}\left[n-{\hat{n}}_{\tau }\right]\;e^{-j2\pi {\hat{f}}_{d}nT_{s}},$$

where $N_{\mathrm{sample}}$ is the number of coherent samples (so that $T_{\mathrm{int}}=N_{\mathrm{sample}}T_{s}$). Define the Doppler error $\Delta f≜f_{d}-{\hat{f}}_{d}$ and the code-phase error $\Delta n_{\tau }≜n_{\tau }-{\hat{n}}_{\tau }$.

Ignoring noise, the correlation can be expressed as

(5) $$R\left(\Delta f,\Delta n_{\tau }\right)=A\sum_{n=0}^{N_{\mathrm{sample}}-1}c\left[n-n_{\tau }\right]\;c\left[n-{\hat{n}}_{\tau }\right]\;e^{j2\pi \Delta fnT_{s}}.$$

Idealized code autocorrelation. For analytical tractability, we adopt an idealized triangular mainlobe model

(6) $$R_{\mathrm{code}}\left(\Delta \tau \right)=\left\{\begin{array}{cc} 1-\frac{\left|\Delta \tau \right|}{T_{c}}, & \left|\Delta \tau \right|\leq T_{c},\\ 0, & |\Delta \tau |>T_{c}, \end{array}\right.$$

where $T_{c}$ is the chip period. This approximation captures the dominant mainlobe behavior used in acquisition analysis.

### 2.3. Frequency Response Characteristics

When the code phase is perfectly aligned ($\Delta n_{\tau }=0$), the correlation reduces to

(7) $$R\left(\Delta f,0\right)=A\sum_{n=0}^{N_{\mathrm{sample}}-1}e^{j2\pi \Delta fnT_{s}}=A\;\cdot \;\frac{1-e^{j2\pi \Delta fN_{\mathrm{sample}}T_{s}}}{1-e^{j2\pi \Delta fT_{s}}}.$$

Applying Euler’s identity, it can be rewritten as [19,20]

(8) $$R\left(\Delta f,0\right)=A\;\frac{\sin\;\left(\pi \Delta fN_{\mathrm{sample}}T_{s}\right)}{\sin\;\left(\pi \Delta fT_{s}\right)}\;e^{j\pi \Delta f\left(N_{\mathrm{sample}}-1\right)T_{s}}.$$

Using $\sin\left(\pi \Delta fT_{s}\right)\approx \pi \Delta fT_{s}$ for small |Δf|, the mainlobe magnitude is approximated by

(9) $$\left|R\left(\Delta f,0\right)\right|\approx A\;N_{\mathrm{sample}}\left|\mathrm{sinc}\;\left(\Delta fT_{\mathrm{int}}\right)\right|,\;\mathrm{sinc}\left(x\right)≜\frac{\sin(\pi x)}{\pi x},$$

where $T_{\mathrm{int}}=N_{\mathrm{sample}}T_{s}$ is the coherent integration time.

#### 2.3.1. Doppler-Bin Coverage and Step Size

The first null of the sinc response occurs at $\left|\Delta f\right|=1/T_{\mathrm{int}}$, which characterizes the mainlobe width. With a Doppler grid spacing $f_{\mathrm{step}}$, the worst-case mismatch between a true Doppler and the nearest grid point is approximately $f_{\mathrm{step}}/2$. Requiring this mismatch to stay within the mainlobe yields

(10) $$\frac{f_{\mathrm{step}}}{2}\leq \frac{1}{T_{\mathrm{int}}}\;⟹\;f_{\mathrm{step}}\leq \frac{2}{T_{\mathrm{int}}}.$$

In practice, a more conservative choice such as $f_{\mathrm{step}}=1/T_{\mathrm{int}}$ is often adopted to limit the worst-case correlation loss.

#### 2.3.2. High-Dynamic Effects

In high-dynamic scenarios, a large Doppler search range (denoted by $f_{d,\max}$) enlarges the search space, and a non-negligible Doppler rate ${\dot{f}}_{d}$ causes the instantaneous Doppler to vary over the coherent interval. Over $T_{\mathrm{int}}$, the total Doppler change is approximately $\Delta f_{d}\approx \left|{\dot{f}}_{d}\right|T_{\mathrm{int}}$, and the maximum mismatch relative to a constant-Doppler replica is on the order of $\Delta f_{d}/2$. When this mismatch becomes comparable to the mainlobe width $O(1/T_{\mathrm{int}})$, the constant-Doppler assumption degrades coherent correlation and impacts acquisition reliability.

### 2.4. Frequency Search Complexity Analysis

Traditional acquisition methods perform an exhaustive, point-by-point search over both Doppler frequency offset and code phase. The total number of search cells can be expressed as

(11) $$N_{\mathrm{cell}}=N_{f}N_{\tau },$$

where $N_{f}$ is the number of Doppler frequency bins and $N_{\tau }$ is the number of code-phase hypotheses.

In high-dynamic scenarios, $N_{f}$ can easily reach several hundred or even several thousand. When combined with a dense code-phase grid $N_{\tau }$, the total search volume $N_{\mathrm{cell}}$ becomes extremely large, resulting in very high computational complexity and making real-time acquisition difficult on resource-constrained platforms.

## 3. Frequency-Domain Acquisition Scheme Based on Compressive Sensing

Compressive sensing (CS) provides a new paradigm for signal acquisition, enabling accurate reconstruction from far fewer measurements than the Nyquist rate requires, provided the underlying signal is sparse or compressible in some transform domain [21,22,23]. When a signal can be represented by only a small number of significant coefficients in an appropriate basis, CS theory guarantees the original high-dimensional signal can be recovered from a small set of linear measurements taken via a carefully designed measurement matrix.

Leveraging this property, the proposed GNSS acquisition algorithm compresses the high-dimensional frequency-domain search space onto a low-dimensional subspace using a structured measurement matrix. By performing sparse reconstruction in this compressed domain, the algorithm effectively reduces the number of Doppler frequency hypotheses that must be searched while preserving acquisition performance. This section introduces the frequency-domain sparse representation of the GNSS signal and the associated compressed measurement and reconstruction scheme.

### 3.1. Frequency-Domain Sparse Representation

First, based on discretized Doppler frequency points, we construct the Doppler frequency-offset vector

(12) $$f={\left[f_{1},f_{2},\ldots ,f_{N_{f}}\right]}^{T},$$

where $N_{f}$ denotes the total number of Doppler frequency bins.

Each Doppler frequency point is linearly spaced as

(13) $$f_{k}=-f_{d,\max}+\left(k-1\right)f_{\mathrm{step}},\;k=1,2,\ldots ,N_{f},$$

where $f_{d,\max}$ is maximum Doppler frequency offset and $f_{\mathrm{step}}$ is the Doppler search step. This construction ensures complete coverage of the Doppler search space from $-f_{d,\max}$ to $f_{d,\max}$.

Next, using the Doppler frequency vector f, construct the frequency-offset basis matrix

(14) $$F=\left[f_{1},\ldots ,f_{N_{f}}\right]\in C^{N_{\mathrm{sample}}\times N_{f}},$$

where $N_{\mathrm{sample}}$ is the number of samples within one coherent integration interval, and each column $f_{k}$ is

(15) $$f_{k}={\left[1,e^{j2\pi f_{k}T_{s}},\ldots ,e^{j2\pi f_{k}\left(N_{\mathrm{sample}}-1\right)T_{s}}\right]}^{T}.$$

Here, $T_{s}$ is sampling interval. Each vector $f_{k}$ represents a complex exponential at Doppler frequency $f_{k}$ over the observation window.

Finally, by circularly shifting the local pseudorandom code vector, construct the complete code-phase search space

(16) $$C=\left[c_{0},\ldots ,c_{N_{\tau }-1}\right]\in R^{N_{\mathrm{sample}}\times N_{\tau }},$$

where $N_{\tau }$ is the number of code-phase (delay) hypotheses, and $c_{i}$ denotes the *sampled* local code replica shifted by *i* samples $\left(i=0,1,\ldots ,N_{\tau }-1\right)$. The corresponding delay is $\tau_{i}=i/f_{s}$. With $f_{s}=2R_{c}$, one chip corresponds to two samples, i.e., a shift of *i* samples equals i/2 chips. In this work, we use an integer-sample delay grid, thus $N_{\tau }=N_{\mathrm{sample}}$ (the number of delay hypotheses within one code period). Each $c_{i}$ is obtained by circularly shifting the sampled local code vector over one code period. In this way, the joint Doppler–code-phase search space is represented by the frequency-offset matrix F and the code-phase matrix C, forming the basis for the subsequent frequency-domain compressive sensing framework [24].

### 3.2. Compressed Measurement and Signal Reconstruction

In the signal reconstruction stage, the joint search over Doppler frequency and code phase is decoupled into a set of independent sparse optimization problems by exploiting frequency-domain sparsity of the received signal. For each code-phase hypothesis *i*, define the ideal frequency-domain vector

(17) $$x_{i}=F^{H}\left(r⊙c_{i}\right)\in C^{N_{f}},$$

where r is the baseband received-signal vector, $c_{i}$ is the local code vector corresponding to the *i*-th code phase, ⊙ denotes the Hadamard product, and ${\left(\;\cdot \;\right)}^{H}$ denotes Hermitian transpose. The vector $x_{i}$ contains frequency-domain coefficients at the discretized Doppler frequency points for code-phase hypothesis *i*.

To reduce dimensionality, construct the compressed observation vector

(18) $$y_{i}=\Phi x_{i}\in C^{M},$$

where $\Phi \in C^{M\times N_{f}}$ is the measurement matrix, and $M\ll N_{f}$ is the number of compressed measurements. The measurement matrix Φ projects the $N_{f}$-dimensional frequency-domain vector $x_{i}$ onto an *M*-dimensional subspace while approximately preserving the information required for sparse reconstruction.

Given compressed observations $y_{i}$, sparse frequency-domain vectors are recovered using the orthogonal matching pursuit (OMP) algorithm [25]:

(19) $${\hat{x}}_{i}=\mathrm{OMP}\left(y_{i},\Phi ,K_{\mathrm{sparse}}\right),\;i=0,1,\ldots ,N_{\tau }-1,$$

where $K_{\mathrm{sparse}}$ denotes the assumed sparsity level of $x_{i}$, and ${\hat{x}}_{i}$ is the reconstructed sparse vector. In a single-satellite scenario, $x_{i}$ is typically one-sparse or contains only a few significant components, corresponding to the true Doppler frequency offset.

Finally, code phase and Doppler frequency offset are jointly estimated via peak detection over all reconstructed sparse vectors ${\hat{x}}_{i}$:

(20) $$\left(i_{0},k_{0}\right)=\arg\max_{i,k}\left|{\hat{x}}_{i}\left(k\right)\right|,$$

where ${\hat{x}}_{i}\left(k\right)$ denotes the *k*-th element of ${\hat{x}}_{i}$. The pair $\left(i_{0},k_{0}\right)$ yields the estimated code phase and Doppler frequency offset, respectively, completing the acquisition process.

### 3.3. Measurement Matrix Optimization Method

#### 3.3.1. Coherence Minimization Objective Function

In compressive sensing theory, the column coherence μ(Φ) of measurement matrix $\Phi \in C^{M\times N_{f}}$ is defined as the maximum absolute value of normalized inner products between any two distinct column vectors [26,27]:

(21) $$\mu \left(\Phi \right)=\max_{1\leq i<j\leq N_{f}}\frac{\left|\langle \phi_{i},\phi_{j}\rangle \right|}{{∥\phi_{j}∥}_{2}{∥\phi_{j}∥}_{2}},$$

where $\phi_{i}$ and $\phi_{j}$ denote the *i*-th and *j*-th column vectors of Φ.

According to the Welch bound [28], for any measurement matrix $\Phi \in C^{M\times N_{f}}$, the coherence admits the theoretical lower bound

(22) $$\mu \left(\Phi \right)\geq \sqrt{\frac{N_{f}-M}{M(N_{f}-1)}}.$$

When Φ forms an equiangular tight frame (ETF) [29], μ(Φ) approaches this lower bound. Therefore, designing an optimal measurement matrix with minimal coherence can be formulated as the following constrained minimax problem [30]:

(23) $$\min_{\Phi }\max_{i\neq j}|G\left(i,j\right)|\;\mathrm{subject}\;\mathrm{to}\;∥\phi_{i}{∥}_{2}=1,\forall i,$$

where $G=\Phi^{H}\Phi \in C^{N_{f}\times N_{f}}$ is the Gram matrix associated with Φ.

Since the optimization problem in (23) is non-smooth and non-convex, directly solving it is challenging. To obtain a differentiable and numerically tractable objective that simultaneously penalizes large off-diagonal Gram matrix entries and enforces unit-norm columns, the following objective function is introduced:

(24) $$J\left(\Phi \right)=∥\Phi^{H}\Phi -G_{\mathrm{target}}{∥}_{F}^{2}+\lambda \sum_{i=1}^{N_{f}}(∥\phi_{i}{{∥}_{2}^{2}-1)}^{2},$$

where $G_{\mathrm{target}}$ is a dynamically adjusted target Gram matrix and λ>0 is a penalty factor constraining the column norms of Φ to remain close to unity, preventing energy imbalance during optimization.

The target Gram matrix $G_{\mathrm{target}}$ is constructed by applying soft-thresholding to the current Gram matrix G under threshold parameter $\tau_{\mathrm{thresh}}$. Denoting the initial threshold as [31]

(25) $$\tau_{\mathrm{thresh},0}=\max_{i\neq j}\left|G_{\mathrm{init}}\left(i,j\right)\right|,$$

the entries of $G_{\mathrm{target}}$ are defined as

(26) $$G_{\mathrm{target}}\left(i,j\right)=\left\{\begin{array}{cc} 1, & i=j,\\ \mathrm{sign}\left(G\left(i,j\right)\right)\max(0,|G\left(i,j\right)|-\tau_{\mathrm{thresh}}), & |G\left(i,j\right)|>\tau_{\mathrm{thresh}},\\ G(i,j), & \left|G\left(i,j\right)\right|\leq \tau_{\mathrm{thresh}}, \end{array}\right.$$

where sign(·) denotes the sign function. For high-coherence elements with $|G\left(i,j\right)|>\tau_{\mathrm{thresh}}$, soft thresholding gradually suppresses large off-diagonal terms. For low-coherence elements with $\left|G\left(i,j\right)\right|\leq \tau_{\mathrm{thresh}}$, the original correlation is preserved to avoid over-shrinking that could lead to ill-conditioned Gram matrices.

#### 3.3.2. Projected Gradient Descent with Two-Stage Annealing Strategy

The objective J(Φ) is minimized using projected gradient descent with momentum and a two-stage annealing schedule.

First, using standard rules of matrix calculus, the gradient of the Frobenius norm satisfies $\frac{{\partial ∥A∥}_{F}^{2}}{\partial A}=2A$ for any matrix A. Applying this identity to each term in (24) yields the gradient of J(Φ) with respect to Φ:

(27) $$\frac{\partial J}{\partial \Phi }=4\Phi \left(\Phi^{H}\Phi -G_{\mathrm{target}}\right)+4\lambda \Phi D,$$

where $D=\mathrm{diag}(∥\phi_{1}{∥}_{2}^{2}-1,\ldots ,∥\phi_{N_{f}}{∥}_{2}^{2}-1)$.

The first term $4\Phi (\Phi^{H}\Phi -G_{\mathrm{target}})$ drives the Gram matrix toward the desired target structure, while the second term 4λΦD regularizes column energies. When $∥\phi_{i}{∥}_{2}^{2}>1$, the corresponding diagonal entry of D is positive, and the gradient pushes $\phi_{i}$ toward smaller norms; conversely, when $∥\phi_{i}{∥}_{2}^{2}<1$, the gradient encourages norm increase, ensuring all columns remain close to the unit sphere.

Given the gradient, a tentative update in the negative gradient direction is

(28) $$\Phi_{\mathrm{temp}}=\Phi -\rho_{k}\frac{\partial J}{\partial \Phi },$$

where $\rho_{k}$ denotes step size at the *k*-th iteration. The step size follows an adaptive decay schedule

(29) $$\rho_{k}=\frac{\rho_{0}}{1+\gamma k},$$

where $\rho_{0}$ is initial step size and γ>0 controls the decay rate. This allows relatively large steps early to accelerate convergence, while gradually reducing step size to stabilize optimization near the optimum.

Since the updated matrix $\Phi_{\mathrm{temp}}$ may violate unit-norm constraints on its columns, it is projected back onto the unit-sphere manifold

(30) $$M=\{\Phi \in C^{M\times N_{f}}:∥\phi_{i}{∥}_{2}=1,\forall i\}$$

via projection operator $P_{M}\left(\;\cdot \;\right)$:

(31) $$\Phi \leftarrow P_{M}\left(\Phi_{\mathrm{temp}}\right),\;\mathrm{where}\;P_{M}\left(\phi_{i}\right)=\frac{\phi_{i}}{∥\phi_{i}{∥}_{2}}.$$

This projection guarantees that all column vectors lie on the unit sphere, and hence, the diagonal entries of the Gram matrix remain equal to one.

Following classical projected gradient descent theory [32], the basic iteration can be written as

(32) $$\Phi^{\left(k+1\right)}=P_{M}(\Phi^{\left(k\right)}-\rho_{k}\nabla J\left(\Phi^{\left(k\right)}\right),$$

where $\nabla J(\Phi^{\left(k\right)})$ denotes the gradient at iteration *k*. Here, $P_{M}\left(\;\cdot \;\right)$ is the projection operator defined above. If additional acceleration is desired, a momentum term can be introduced:

(33) $$\begin{array}{cc} V^{\left(k+1\right)} & =\beta V^{\left(k\right)}+\left(1-\beta \right)\nabla J\left(\Phi^{\left(k\right)}\right), \end{array}$$

(34) $$\begin{array}{cc} \Phi^{\left(k+1\right)} & =P_{M}\left(\Phi^{\left(k\right)}-\rho_{k}V^{\left(k+1\right)}\right), \end{array}$$

where $V^{\left(k\right)}$ is the momentum term and β is the momentum coefficient. The momentum term accumulates the gradient history, and the update direction $V^{\left(k+1\right)}$ is then used in place of the instantaneous gradient to smooth the optimization trajectory and mitigate oscillations in ill-conditioned regions of the objective landscape.

Finally, we employ a two-stage annealing strategy to adaptively adjust the soft-threshold parameter $\tau_{\mathrm{thresh}}$.

Stage One.

In the first stage, $\tau_{\mathrm{thresh}}$ decays exponentially:

(35) $$\tau_{\mathrm{thresh},k}=\tau_{\mathrm{thresh},0}\;\exp\;\left(-2.0\;\frac{k-1}{I_{1}-1}\right),\;k=1,\ldots ,I_{1},$$

where $\tau_{\mathrm{thresh},0}≜\max_{i\neq j}\left|G_{\mathrm{init}}\left(i,j\right)\right|$ is the initial threshold and $I_{1}$ is the number of iterations in the first stage. This rapid decay aggressively suppresses large off-diagonal Gram entries early in the optimization.

Stage Two. In the second stage, the threshold is clamped to a small constant floor:

(36) $$\tau_{\mathrm{thresh},k}=\tau_{\mathrm{thresh},\min},\;k=I_{1}+1,\ldots ,I_{\mathrm{tot}}.$$

with

(37) $$\tau_{\mathrm{thresh},\min}=\max\;\left\{\frac{\log N_{f}}{M},\;\tau_{\mathrm{floor}}\right\}.$$

Since $\tau_{\mathrm{thresh},\min}=\max\left\{\log\left(N_{f}\right)/M,\tau_{\mathrm{floor}}\right\}$, its numerical value depends on the matrix dimensions $\left(M,N_{f}\right)$ and the chosen floor $\tau_{\mathrm{floor}}$ used to stabilize late-stage refinement. Therefore, different $\left(M,N_{f}\right)$ settings may exhibit different plateau levels and convergence trajectories. This low, nearly constant threshold enables fine-scale adjustment of the Gram matrix structure while avoiding excessive shrinkage of small off-diagonal entries.

The projected gradient descent procedure generates an iterative sequence $\{\Phi^{\left(k\right)}\}\subset M$. On regions where the objective is differentiable, the gradient is locally Lipschitz with a Lipschitz constant upper-bounded by the spectral norm of the Hessian,

(38) $$L≜{∥\nabla^{2}J∥}_{2}.$$

Although the overall objective is nonconvex and nonsmooth, the projected gradient updates provide a computationally efficient heuristic and empirically converge to a stable stationary point in our experiments [33].

#### 3.3.3. Algorithm Execution Steps

**Figure 1:**
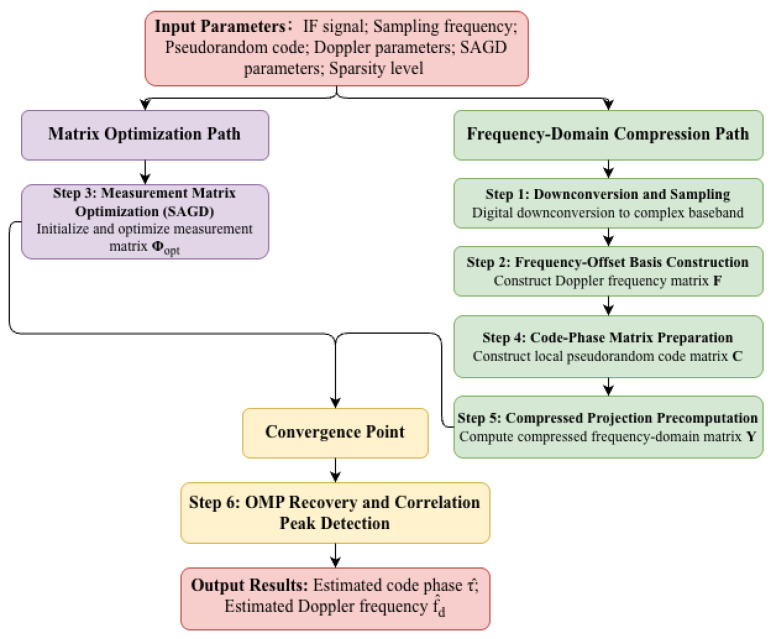
SAGD acquisition algorithm.

The detailed and straightforward processes of proposed acquisition algorithm are shown in Algorithm 1 and Figure 1.


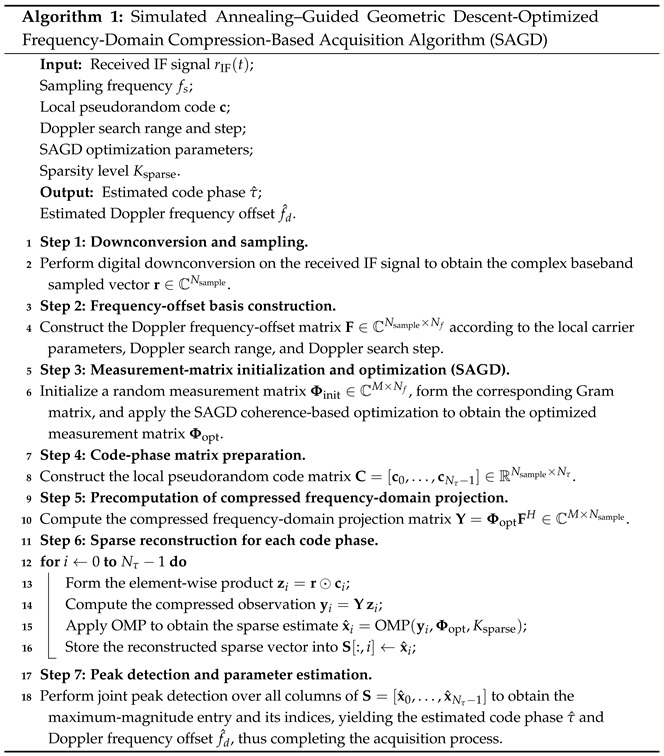


## 4. Algorithm Performance Analysis

The following analysis adopts simplified engineering models to capture the dominant trends in detection performance, rather than exact closed-form performance bounds. The OMP error bounds and measurement-number relations are derived from random measurement matrix theory.

Section 4 provides an engineering-oriented interpretation of why the proposed low-coherence sensing operator improves acquisition. Specifically, Section 4.1 links Doppler uncertainty to sparsity in the discretized frequency dictionary and uses standard OMP recovery intuition to derive how the required measurement number scales with $\left(N_{f},K_{\mathrm{sparse}}\right)$. Section 4.2 then models the acquisition decision event as a competition between the true correlation peak and the maximum spurious peak, and shows how Gram matrix coherence affects the effective SNR and the extreme-value statistics of incorrect peaks. These results are used to guide the choice of compression ratio and to interpret the simulation trends reported in Section 5.

### 4.1. Sparsity Analysis

Assume that the received signal is

(39) $$r=\alpha \;\cdot \;c_{\tau }⊙e^{j2\pi f_{d}t}+w,$$

where $c_{\tau }$ is the spreading-code sequence corresponding to delay τ, $w∼\mathrm{CN}(0,\sigma_{w}^{2}I_{N_{\mathrm{sample}}})$ is complex Gaussian white noise (front-end channel noise), α is the signal amplitude, $t={\left[t_{0},t_{1},\ldots ,t_{N_{\mathrm{sample}}-1}\right]}^{T}$ is the time vector with $t_{n}=n\;\cdot \;T_{s}$ for $n=0,1,\ldots ,N_{\mathrm{sample}}-1$, where $T_{s}$ is the sampling interval, and ⊙ denotes the Hadamard (element-wise) product.

To reduce the dimensionality of signal processing, the proposed algorithm uses a measurement matrix $\Phi \in C^{M\times N_{f}}$ to compress the Doppler-frequency dimension. The measurement matrix is applied to compress and project the frequency-offset matrix $F\in C^{N_{\mathrm{sample}}\times N_{f}}$, forming the precomputed compressed frequency-domain transform matrix

(40) $$Y=\Phi F^{H}\in C^{M\times N_{\mathrm{sample}}},$$

which linearly combines the $N_{f}$ Doppler-frequency basis vectors through Φ to generate *M* combined basis vectors. In this way, the algorithm avoids computing the correlation values for all $N_{f}$ frequency points one by one.

To obtain the code-phase and Doppler-frequency information, sparse reconstruction of the signal must be performed. For each code phase *i*, define the ideal (noiseless) frequency-domain vector $x_{i}=F^{H}\left(r⊙c_{i}\right)\in C^{N_{f}}$, form the observation vector $y_{i}=\Phi x_{i}\in C^{M}$ (with additive measurement noise $n∼\mathrm{CN}(0,\sigma_{n}^{2}I_{M})$), and solve the optimization problem

(41) $$\min_{x_{i}}∥x_{i}{∥}_{0}\;s.t.\;{∥y_{i}-\Phi x_{i}∥}_{2}\leq \varepsilon ,$$

for $i=0,\ldots ,N_{\tau }-1$, where ${\hat{x}}_{i}$ denotes the reconstructed (estimated) sparse vector.

According to compressive sensing theory [21,22,34], when the restricted isometry property (RIP) is satisfied, the OMP reconstruction error obeys

(42) $$∥{\hat{x}}_{i}-x_{i}{∥}_{2}\leq C_{1}^{\mathrm{OMP}}\frac{{∥n∥}_{2}}{\sqrt{M}}+C_{2}^{\mathrm{OMP}}\sigma_{K_{\mathrm{sparse}}}\left(x_{i}\right),$$

where $x_{i}$ is the ideal frequency-domain sparse vector, ${\hat{x}}_{i}$ is the reconstructed vector, n is the measurement noise, and $\sigma_{K_{\mathrm{sparse}}}\left(x_{i}\right)$ denotes the best $K_{\mathrm{sparse}}$-term approximation error with $K_{\mathrm{sparse}}$ equal to the signal sparsity level. The constants $C_{1}^{\mathrm{OMP}}$ and $C_{2}^{\mathrm{OMP}}$ are positive and depend on the restricted isometry constant (RIC) of the measurement matrix. It should be noted that this error bound is derived under the assumption of random measurement matrices.

The classical OMP error bounds and RIP-related results are primarily established for i.i.d. random measurement matrices. In this work, Φ is deterministically obtained after SAGD optimization and thus does not admit a formal random-matrix guarantee. The purpose of invoking these results is to provide engineering insight into dominant scaling trends. Specifically, when Φ is column-normalized and its mutual coherence is substantially reduced (compared with common random/structured baselines), the recovery behavior is mainly governed by the conditioning/coherence of the sensing operator. Under these conditions, random-matrix-inspired expressions can serve as useful approximations for how recovery robustness varies with *M* and $K_{\mathrm{sparse}}$, but they should not be interpreted as strict guarantees for the optimized deterministic Φ.

Although the low-coherence matrix constructed in this paper is not strictly random, its Gram matrix spectral characteristics are similar to those of random matrices satisfying RIP.

According to the Donoho–Tanner theory [35,36], the number of measurements required for successful OMP recovery satisfies

(43) $$M_{\mathrm{crit}}=c_{\mathrm{DT}}\;\cdot \;K_{\mathrm{sparse}}\log\left(\frac{N_{f}}{K_{\mathrm{sparse}}}\right),$$

where $c_{\mathrm{DT}}$ is a positive constant (Donoho–Tanner) and $K_{\mathrm{sparse}}$ is the sparsity level. In GNSS single-satellite single-path scenarios, $K_{\mathrm{sparse}}=1$, so

(44) $$M_{\mathrm{crit}}\approx c_{\mathrm{DT}}\log N_{f},$$

which indicates that the required number of compressed measurements grows only logarithmically with the number of Doppler-frequency bins.

### 4.2. Detection Probability Analysis

#### 4.2.1. Extreme-Value Detection Model

We model acquisition as an extreme-value detection problem using the correlation statistics computed in the matching step of OMP. Let $i_{0}$ denote the true Doppler-bin index. Without loss of generality, we re-index the Doppler bins such that the true bin is $i_{0}=0$. Define

(45) $$c_{i}≜\phi_{i}^{H}y,\;X_{i}≜\left|c_{i}\right|,\;i=0,1,\ldots ,N_{f}-1.$$

The maximum spurious peak is

(46) $$X_{\max}≜\max_{i\in \{1,\ldots ,N_{f}-1\}}X_{i}.$$

The detection probability is

(47) $$P_{d}≜P\;\left(X_{0}>X_{\max}\right),$$

i.e., the probability that the true peak exceeds the largest spurious peak among the $N_{f}-1$ competitors.

#### 4.2.2. Role of Coherence

Assuming unit-norm columns $∥\phi_{i}{∥}_{2}=1$, the (mutual) coherence is defined as

(48) $$\mu ≜\max_{i\neq j}\left|\phi_{i}^{H}\phi_{j}\right|.$$

A smaller μ reduces inter-column leakage, thereby lowering the mean and variance of the incorrect-peak statistics ${\left\{X_{i}\right\}}_{i\neq i_{0}}$ and improving peak separability.

The analytical expressions below are engineering approximations. Under sufficiently low coherence, the incorrect-position statistics are treated as approximately i.i.d., and an empirical extreme-value model is used to approximate the distribution of $X_{\max}$ for tractable performance prediction.

#### 4.2.3. Single-Sparse Signal Model

Consider a single-sparse signal

(49) $$s=\alpha e_{i_{0}}.$$

where $e_{i_{0}}$ is the standard basis vector and α is signal amplitude, noise $n∼\mathrm{CN}(0,\sigma_{n}^{2}I_{M})$ and form compressed measurements

(50) $$y=\Phi s+n=\alpha \phi_{i_{0}}+n.$$

where $\phi_{i_{0}}$ is the $i_{0}$-th column of measurement matrix Φ.

The Gram matrix coherence $\mu =\max_{i\neq j}\left|\phi_{i}^{H}\phi_{j}\right|$ reflects maximum correlation between columns of Φ. During OMP iteration, the correlation computation yields the correct-position correlation value

(51) $$X_{0}=\left|\alpha +\phi_{i_{0}}^{H}n\right|.$$

and incorrect-position correlation values ($i\neq i_{0}$)

(52) $$X_{i}=\left|\alpha \;\phi_{i}^{H}\phi_{i_{0}}+\phi_{i}^{H}n\right|,\;i\neq i_{0}.$$

where $X_{0}$ denotes correlation at the true signal position $i_{0}$ and $X_{i}$ denotes correlations at other positions *i*.

Since $G=\Phi^{H}\Phi$, we have

(53) $$\left|\phi_{i}^{H}\phi_{i_{0}}\right|=\left|G\left(i,i_{0}\right)\right|\leq \mu .$$

Thus, the mean of the incorrect peaks can be approximated as

(54) $$E[X_{i}]\approx |\alpha |\;\mu .$$

From the compressed measurement model $y=\alpha \phi_{i_{0}}+n$ and the column normalization condition $∥\phi_{i_{0}}{∥}_{2}=1$, the total energy of the signal component in the measurement space is ${\left|\alpha \right|}^{2}$. This energy is distributed over the entries of $\phi_{i_{0}}$ and satisfies $\sum_{m=1}^{M}{\left|\phi_{i_{0}}\left(m\right)\right|}^{2}=1$.

After noise projection, using the column normalization condition $∥\phi_{i_{0}}{∥}_{2}=1$ and the statistical independence of the noise $n∼\mathrm{CN}(0,\sigma_{n}^{2}I_{M})$, the noise variance at each measurement point remains $\sigma_{n}^{2}$.

From the above analysis, column normalization keeps the per-component noise variance at $\sigma_{n}^{2}$. From an intuitive dimensionality-reduction perspective, if the original frequency-domain noise is i.i.d. with dimension $N_{f}$, its total energy is $N_{f}\sigma_{n}^{2}$; after compression, the noise dimension is reduced to *M*, and the total noise energy becomes $M\sigma_{n}^{2}$. Thus, although compressed projection does not change the single-point SNR, detection performance is still primarily determined by the coherence μ.

At the same time, it is necessary to control the leakage of energy between columns. For $i\neq i_{0}$, the off-diagonal Gram matrix elements $G\left(i,i_{0}\right)=\phi_{i}^{H}\phi_{i_{0}}$ are bounded by the coherence μ, i.e., $|G(i,i_{0})|\leq \mu$. Reducing μ weakens inter-column leakage and thereby suppresses interference at incorrect positions. Coherence μ introduces both bias and increased variance, and these two effects jointly deteriorate the detectability of the correct signal.

#### 4.2.4. Effective Signal-to-Noise Ratio (SNR) Analysis

Assume that the original uncompressed frequency-domain SNR per frequency bin is

(55) $$\mathrm{SNR}=\frac{{\left|\alpha \right|}^{2}}{\sigma_{n}^{2}}.$$

where ${\left|\alpha \right|}^{2}$ is the signal power at the correct frequency bin and $\sigma_{n}^{2}$ is the noise power per frequency bin.

When the Gram matrix coherence is μ, the correct peak suffers both signal attenuation and increased leakage from incorrect peaks. The corresponding effective SNR can be modeled as

(56) $${\mathrm{SNR}}_{\mathrm{eff}}=\frac{{\left|\alpha \right|}^{2}\;N_{f}\;{\left(1-a_{1}\mu \right)}^{2}}{\sigma_{n}^{2}+a_{2}\;{\left|\alpha \right|}^{2}\;N_{f}\;\mu^{2}}.$$

where $a_{1}\approx 1$ represents the signal-power attenuation factor and $a_{2}\approx$ 0.5∼1 represents the enhancement factor associated with interference leakage at incorrect positions. Note that $a_{1}$ and $a_{2}$ are empirical SNR model coefficients distinct from the OMP error-bound constants $C_{1}^{\mathrm{OMP}}$ and $C_{2}^{\mathrm{OMP}}$ in (42). This expression shows that even though compressed projection does not change the per-component SNR, the coherence μ still influences detection performance by reducing the effective SNR.

#### 4.2.5. Detection Probability Extreme Value Distribution Model

Define $X_{\max}$ as in (46) and let $Z≜X_{\max}$. Then $P_{d}=P\left(X_{0}>Z\right)$, where $X_{0}$ follows a Rice distribution and *Z* is the maximum of the $N_{f}-1$ incorrect-peak magnitudes.

According to extreme-value statistical theory [37,38], when the sample size is large, the incorrect-position correlation values can be approximated as independent and identically distributed random variables. Under this assumption, an engineering approximation for the distribution of the maximum is adopted. Let $Z=\max_{i\neq i_{0}}X_{i}$; when $N_{f}\gg 1$, an empirical extreme-value model based on the Gumbel distribution is used [39], with parameters obtained by matching the mean and variance. The cumulative distribution function of the maximum incorrect peak *Z* is approximated as

(57) $$F_{Z}\left(z\right)=\exp\;\left[-\left(N_{f}-1\right)\exp\;\left(-\frac{{\left(z-\mu_{Z}\right)}^{2}}{2\sigma_{Z}^{2}}\right)\right],$$

where $\mu_{Z}=\left|\alpha \right|\mu +\sigma_{n}\sqrt{2\ln(N_{f}-1)}$, taking into account the residual correlations between the Gram matrix column vectors, the variance of the incorrect peak is modeled as

(58) $$\sigma_{Z}^{2}=\sigma_{n}^{2}+{\left|\alpha \right|}^{2}\mu^{2}\left(1+a_{3}\left(N_{f}-2\right)\right).$$

with $a_{3}$ an engineering fitting parameter that reflects the cumulative effect of residual inter-column correlations. When the column vectors are strictly orthogonal, $a_{3}\to 0$; when the coherence is large, $a_{3}$ approaches its upper bound.

The detection event corresponds to $X_{0}>Z$, and the detection probability is given by (57). Combining the Rice distribution of $X_{0}$ with the empirical extreme-value model of *Z* yields the integral expression

(59) $$P_{d}=\int_{0}^{\infty }f_{X_{0}}\left(x\right)\;F_{Z}\left(x\right)\;dx.$$

where $f_{X_{0}}\left(x\right)=\frac{2x}{\sigma_{n}^{2}}\exp\left(-\frac{x^{2}+{\left|\alpha \right|}^{2}}{\sigma_{n}^{2}}\right)I_{0}\left(\frac{2x|\alpha |}{\sigma_{n}^{2}}\right)$ is the Rice probability density function [40], and $I_{0}\left(\;\cdot \;\right)$ is the modified Bessel function of the first kind of order zero. Thus, the full expression becomes

(60) $$P_{d}=\int_{0}^{\infty }\frac{2x}{\sigma_{n}^{2}}\exp\;\left(-\frac{x^{2}+{\left|\alpha \right|}^{2}}{\sigma_{n}^{2}}\right)I_{0}\;\left(\frac{2x|\alpha |}{\sigma_{n}^{2}}\right)\;\cdot \;\exp\;\left[-\left(N_{f}-1\right)\exp\;\left(-\frac{{\left(x-\mu_{Z}\right)}^{2}}{2\sigma_{Z}^{2}}\right)\right]\;dx.$$

which provides an approximate analytical characterization of the detection probability under the influence of Gram matrix coherence.

This expression reflects the impact of coherence μ on detection performance.

#### 4.2.6. Monte Carlo Estimation

Since the integral-form expression in (59) and (60) is a theoretical expression that is difficult to evaluate analytically, Monte Carlo simulation is typically employed to estimate the detection probability [41]:

(61) $$P_{d}\approx \frac{1}{N_{\mathrm{MC}}}\sum_{i=1}^{N_{\mathrm{MC}}}I\;\left(X_{0}^{\left(i\right)}>\max_{j\neq i_{0}}X_{j}^{\left(i\right)}\right).$$

where $N_{\mathrm{MC}}=10^{4}∼10^{5}$ is sufficient to obtain stable and reliable Monte Carlo estimates, and ⊮(·) is the indicator function.

## 5. Simulation Validation

### 5.1. Simulation Parameter Settings

We use the GPS L1 signal as an example with $R_{b}=50$ bps, $R_{c}=1.023$ MHz, and $f_{s}=2R_{c}$. The Doppler dictionary contains $N_{f}=256$ frequency bins, and the compressed measurement length is M=192. To characterize high dynamics, we consider a wide Doppler uncertainty range $f_{d}\in \left[-128,128\right]$ kHz, which leads to a large Doppler search volume in conventional acquisition. In addition, we evaluate Doppler-rate effects using a time-varying Doppler model $f_{d}\left(t\right)=f_{d,0}+{\dot{f}}_{d}t$ and sweep ${\dot{f}}_{d}$ over 0–1000 Hz/s.

### 5.2. Simulation Objectives

**Figure 2:**
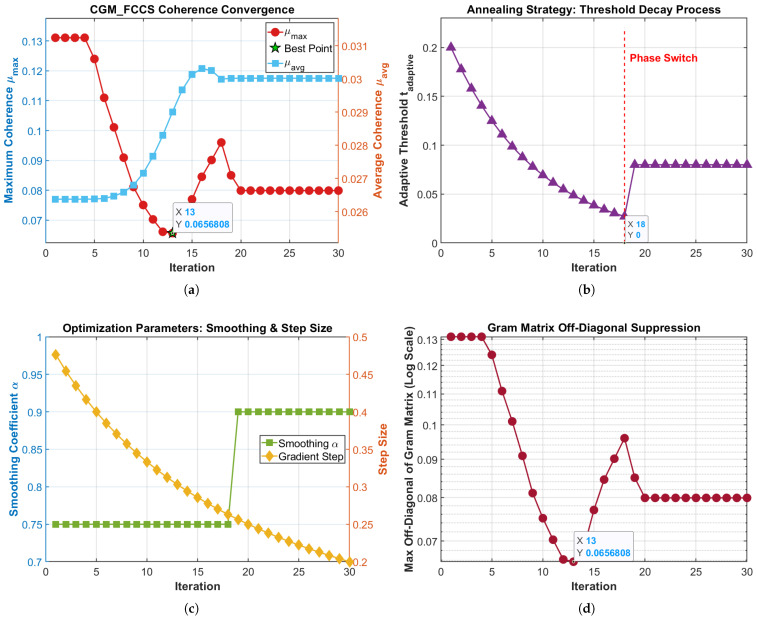
Convergence behavior of the proposed SAGD measurement-matrix optimization: (**a**) evolution of maximum and average coherence; (**b**) two-stage threshold schedule; (**c**) scheduling of key optimization parameters; (**d**) suppression of the dominant off-diagonal Gram component.

**Figure 3:**
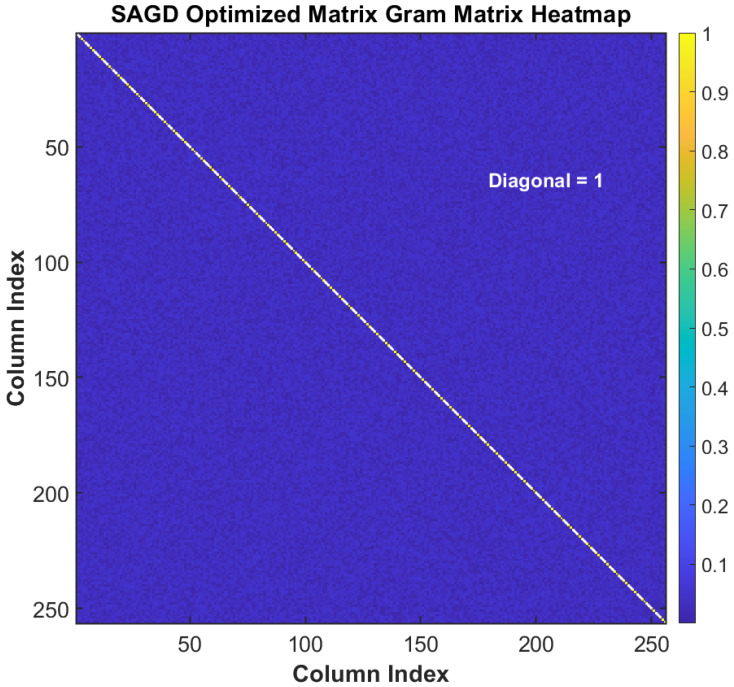
Heatmap of the Gram matrix $G=\Phi^{H}\Phi$ for the SAGD-optimized sensing operator.

**Figure 4:**
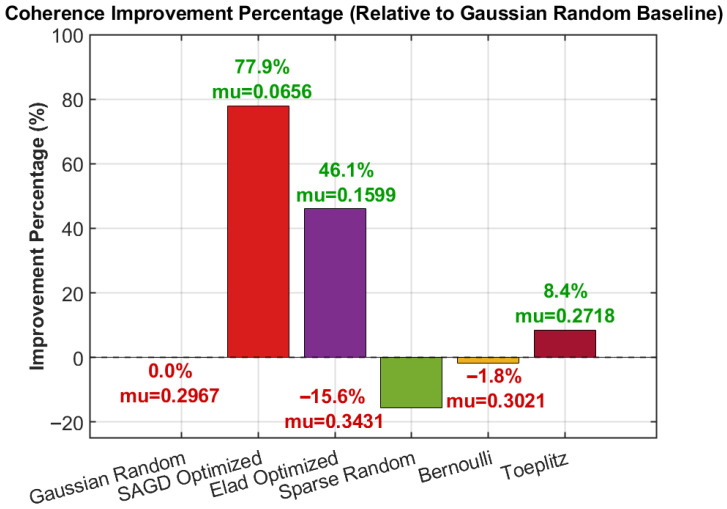
Coherence improvement percentage (relative to the Gaussian-random baseline) for different measurement-matrix designs.

**Figure 5:**
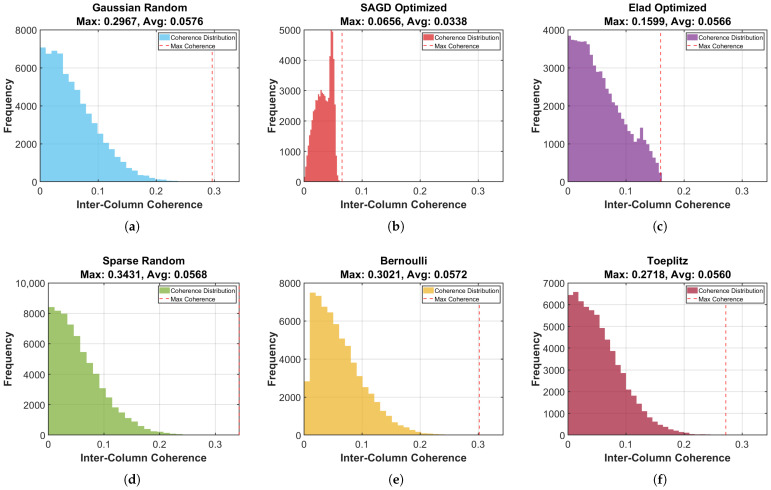
Inter-column coherence distribution comparison for different measurement matrices.

**Figure 6:**
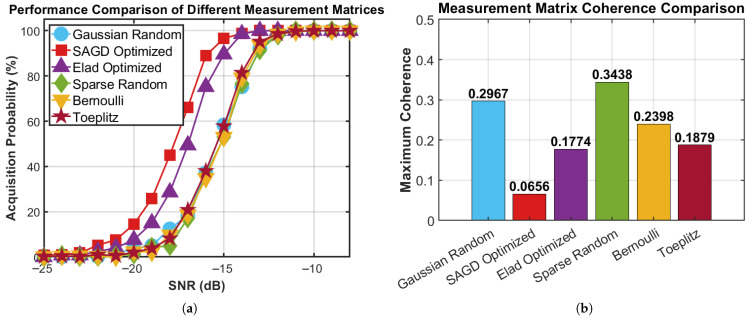
Acquisition probability vs. SNR in the FCS framework: (**a**) acquisition probability versus SNR; **(b**) coherence of the corresponding measurement matrices.

**Figure 7:**
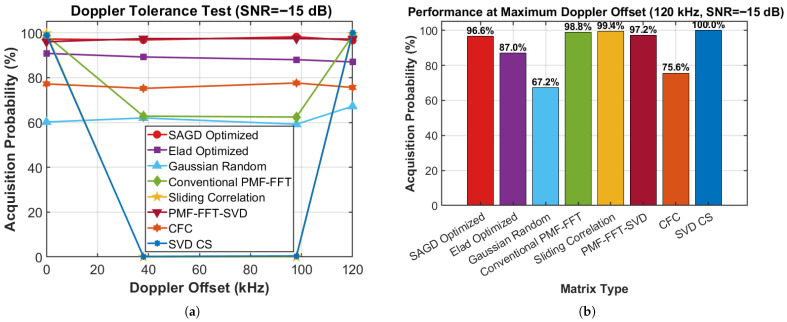
Doppler-offset tolerance performance comparison: (**a**) acquisition probability versus Doppler offset; (**b**) comparison at the maximum tested Doppler offset.

**Figure 8:**
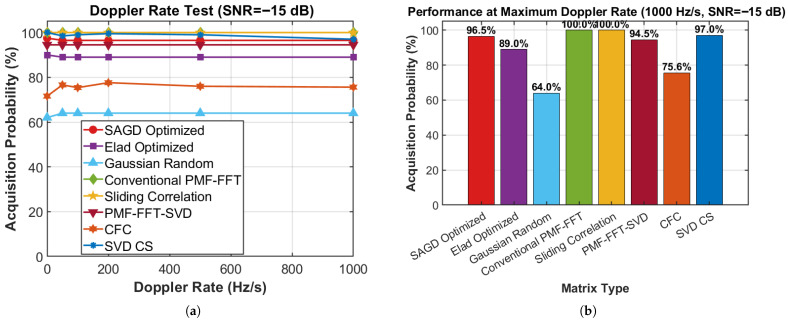
Doppler-rate robustness comparison: (**a**) acquisition probability versus Doppler rate; (**b**) comparison at the maximum tested Doppler rate.

**Figure 9:**
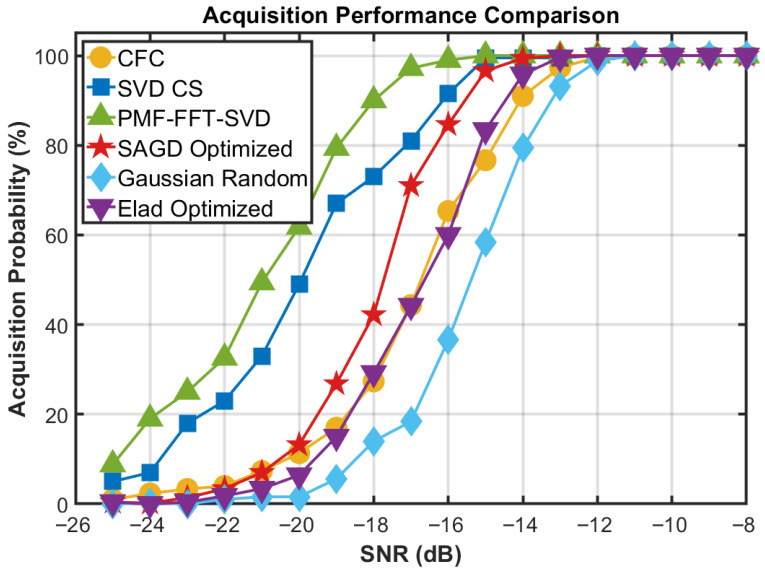
Overall acquisition-performance comparison against representative acquisition schemes discussed in Section 1.

**Figure 10:**
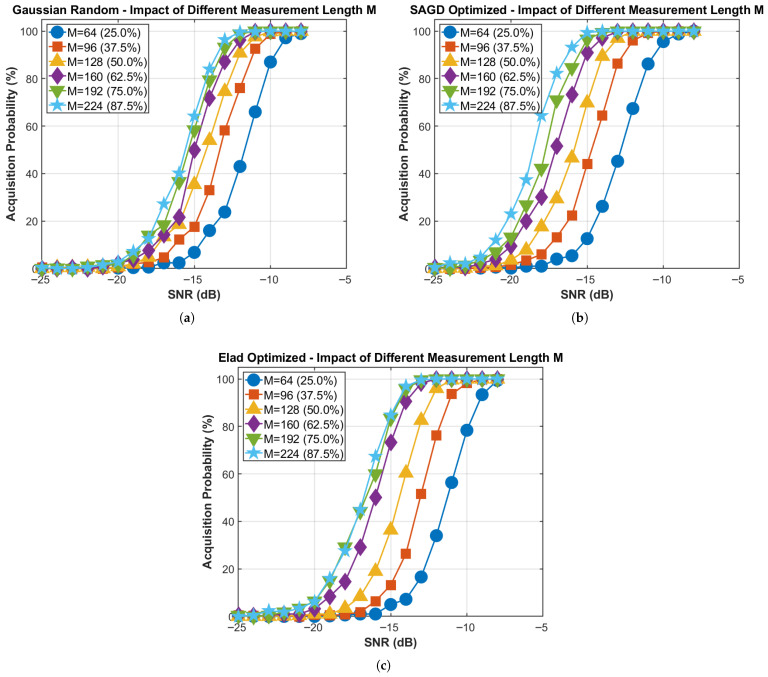
Impact of measurement length *M* (compression ratio) for three acquisition methods. (**a**) Gaussian random: impact of *M*; (**b**) SAGD optimized: impact of *M*; (**c**) Elad optimized: impact of *M*.

**Figure 11:**
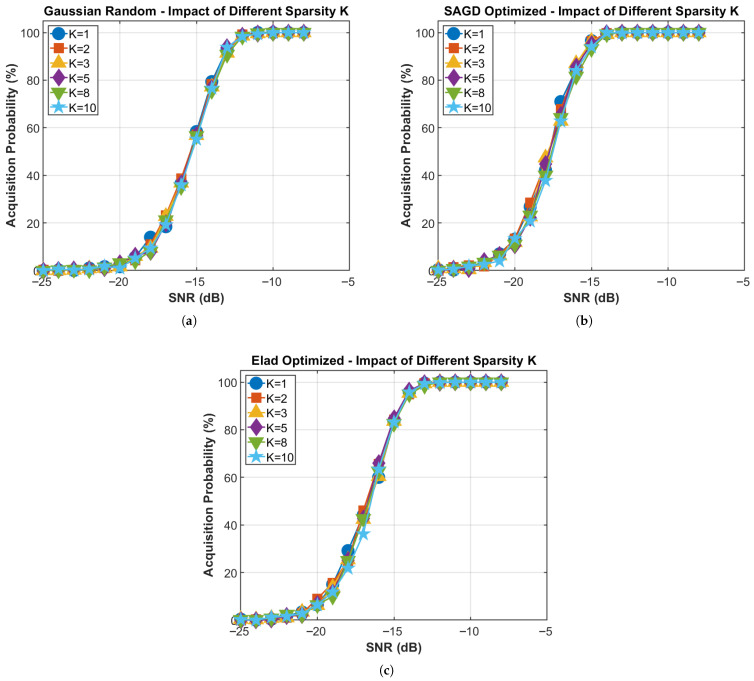
Impact of sparsity $K_{\mathrm{sparse}}$ on acquisition performance. (**a**) Gaussian random: impact of $K_{\mathrm{sparse}}$; (**b**) SAGD optimized: impact of $K_{\mathrm{sparse}}$; (**c**) Elad optimized: impact of $K_{\mathrm{sparse}}$.

This section validates the proposed SAGD-optimized frequency-domain compressive acquisition from three complementary perspectives. First, we verify that SAGD produces a low-coherence sensing operator by analyzing convergence behavior and Gram/coherence statistics (Figure 2, Figure 3, Figure 4 and Figure 5). Second, we quantify weak-signal sensitivity through acquisition-probability curves versus SNR within the same frequency compressed sensing (FCS) pipeline (Figure 6). Third, to substantiate practical benefits for resource-constrained receivers, we report measured online CPU time under the same software/hardware settings (Table 1) and discuss the associated memory/computation implications. Finally, we evaluate robustness under high dynamics by sweeping both Doppler offset and Doppler rate (Figure 7 and Figure 8), position the proposed method against the representative literature baselines (Figure 9), and study sensitivity to compression ratio and sparsity assumptions (Figure 10 and Figure 11).

### 5.3. Online Runtime Metric

To quantify practical engineering benefit, we measure the online wall-clock CPU time of each acquisition scheme on the same test bed (Table 2) and report the results in Table 2. Here, “online” refers to the real-time processing path executed per acquisition attempt. For SAGD–FCS, the reported time covers forming compressed observations (including the projection using a fixed $\Phi_{\mathrm{opt}}$ and the corresponding low-dimensional statistics) and the subsequent sparse recovery/peak decision; the SAGD matrix-design procedure is performed offline and is not counted in online latency.

As shown in Table 2, the proposed SAGD–FCS achieves an online CPU time of 0.077227 s, corresponding to a 17.86× speedup over sliding-correlation acquisition (1.379280 s), a 10.47× speedup over PMF–FFT–SVD (0.808612 s), and a 1.77× speedup over the CFC baseline (0.137015 s). Within the same FCS pipeline, SAGD–FCS is 1.24× faster than Gaussian-random FCS and 1.22× faster than Elad-optimized FCS. These results substantiate that the proposed approach reduces online computational burden in practice.

The main memory footprint of SAGD–FCS comes from storing $\Phi_{\mathrm{opt}}\in C^{M\times N_{f}}$ and (optionally) the projection operator used for fast online measurement formation. With M=192 and $N_{f}=256$, $\Phi_{\mathrm{opt}}$ contains 192×256=49,152 complex entries (≈0.75 MB in double-complex storage). With $f_{s}=2R_{c}$ and $T_{\mathrm{int}}=1$ ms, $N_{\mathrm{sample}}=f_{s}T_{\mathrm{int}}=2046$, hence *Y* contains 192×2046=392,832 complex entries (≈6.3 MB in double-complex storage). In practice, *Y* can be stored in single precision or computed in blocks if peak memory is constrained. Since all schemes are benchmarked on the same platform, the online CPU time also serves as a practical proxy for relative CPU-cycle consumption and thus indicates the relative energy/load trend under comparable operating conditions.

### 5.4. SAGD Optimization and Matrix Statistics

We first visualize the SAGD optimization trajectory and the resulting Gram coherence statistics to confirm that the proposed method suppresses inter-column leakage, which is one of the core principles behind robust sparse recovery in compressed acquisition.

To explicitly demonstrate how SAGD reshapes the sensing operator, we visualize the evolution of the coherence metrics and the associated hyper-parameter schedule, which clarifies the role of the two-stage annealing strategy. As shown in Figure 2a, the maximum coherence decreases rapidly during the early iterations and may exhibit a mild rebound before stabilizing. The deployed sensing matrix is selected by the best-iteration criterion, i.e., $\Phi_{\mathrm{opt}}=\Phi^{\left(k^{★}\right)}$ with $k^{★}=\arg\min_{k}\mu_{\max}^{\left(k\right)}$, rather than using the last iterate. During Stage 1, the soft-threshold parameter decays exponentially (Figure 2b), which aggressively suppresses large off-diagonal Gram entries and accelerates coherence reduction, but can over-regularize the off-diagonal structure and cause a temporary increase in $\mu_{\max}$. Stage 2 clamps the threshold to a constant value $\tau_{\mathrm{thresh},\min}$ and adjusts the optimization parameters (Figure 2c) to improve stability and refine the Gram structure, thereby avoiding late-stage oscillations. Note that the Stage-2 plateau value in Figure 2b equals $\tau_{\mathrm{thresh},\min}$; its definition and dependence on $\left(M,N_{f}\right)$ and $\tau_{\mathrm{floor}}$ are given in Section 3.3.2 (see Equation (37)). Figure 2d shows that the largest off-diagonal Gram entry closely tracks $\mu_{\max}$, confirming that $\mu_{\max}$ is an effective proxy for dominant inter-column leakage. We also plot the average coherence to monitor global energy redistribution during optimization; it remains small and varies mildly, indicating that SAGD suppresses dominant off-diagonal components without inducing numerical instability.

Figure 3 visualizes the Gram matrix $G=\Phi^{H}\Phi$ of the SAGD-optimized sensing operator. The diagonal entries are exactly unity (mean/min/max =1.000000), confirming strict column normalization. Among the $N_{f}\left(N_{f}-1\right)=65280$ off-diagonal entries, the maximum magnitude is 0.0656808, which equals the maximum mutual coherence $\mu_{\max}$. Moreover, the off-diagonal distribution is concentrated at relatively small values: the mean is 0.033902, the median is 0.035084, and the standard deviation is 0.014022. The 95th and 99th percentiles are 0.052811 and 0.055670, respectively. Finally, 60.35% of the off-diagonal entries exceed 0.03, while only 13.11% exceed 0.05. These statistics provide quantitative evidence that SAGD suppresses dominant inter-column leakage and yields a Gram matrix with more concentrated energy on the diagonal and reduced off-diagonal magnitudes.

Figure 4 quantifies the maximum-coherence improvement relative to the Gaussian-random baseline. The proposed SAGD design achieves the largest improvement, reducing the maximum coherence to the lowest value among all candidates. In comparison, structured (e.g., Toeplitz) and alternative optimization baselines provide only moderate or marginal improvements, while sparse-random/Bernoulli constructions may even degrade the worst-case coherence depending on the realization. These results corroborate that the SAGD optimization effectively suppresses dominant inter-column leakage.

As shown in Figure 5, the SAGD-optimized matrix yields a visibly narrower and lower-valued coherence distribution compared with common random/structured constructions, consistent with the Gram-structure evidence in Figure 3. The concentration of off-diagonal Gram entries at smaller magnitudes indicates reduced leakage, which helps improve peak separability in the compressed-domain acquisition decision.

Figure 6 compares six measurement matrices within the FCS framework. The corresponding maximum-coherence values are as follows: Gaussian random ($\mu_{\max}=0.296703$), SAGD optimized ($\mu_{\max}=0.0656808$), Elad optimized ($\mu_{\max}=0.159879$), sparse random ($\mu_{\max}=0.343128$), Bernoulli ($\mu_{\max}=0.302083$), and Toeplitz ($\mu_{\max}=0.271811$). Among all candidates, the proposed SAGD design achieves the lowest coherence, representing a 77.89% reduction relative to the Gaussian baseline. From a quantitative acquisition perspective, the proposed SAGD matrix attains the best weak-signal sensitivity within the FCS pipeline: the SNR required to reach a 90% acquisition probability is −15 dB for SAGD, whereas the corresponding thresholds are −14 dB for Elad and −13 dB for Gaussian random, sparse random, Bernoulli, and Toeplitz designs. At SNR=−16 dB, SAGD yields an acquisition probability of 89.0%, while the Gaussian and Elad matrices achieve only 37.2% and 75.2%, respectively. These results provide clear numerical evidence that coherence-optimized sensing substantially improves peak separability and acquisition reliability in the compressed domain.

### 5.5. High-Dynamic Robustness: Doppler Offset and Doppler Rate

Large Doppler uncertainty (offset) and acceleration-induced Doppler variation (rate) are two key manifestations of high-dynamic acquisition conditions. Therefore, we evaluate robustness by sweeping Doppler offset and Doppler rate under the same SNR and integration settings.

Figure 7a reports acquisition probability versus Doppler offset at SNR=−15 dB. Across the tested offsets {0,38,98,120} kHz, the proposed SAGD–FCS remains highly stable, achieving 97.2%, 96.8%, 98.2%, and 96.6%, respectively (variation within 1.6 percentage points). In contrast, several baselines exhibit pronounced sensitivity to Doppler offset: CFC achieves only 75.2–77.6%, while Gaussian-random CS remains at 59.2–67.2%. The conventional PMF–FFT method and sliding-correlation acquisition perform well at the edge offsets (near 0 and 120 kHz), but degrade severely in the mid-offset region: PMF–FFT drops to 62.4–62.8% and sliding correlation fails (0%) at 38 and 98 kHz. Notably, SVD-CS exhibits strong offset-dependent instability, achieving 99.0% and 100.0% at 0 and 120 kHz but collapsing to 0.2–0.4% at 38 and 98 kHz, indicating grid-mismatch/structure-induced sensitivity under large Doppler uncertainty. Figure 7b summarizes the performance at the maximum tested offset 120 kHz: SAGD achieves 96.6%, outperforming Elad (87.0%), Gaussian random (67.2%), and CFC (75.6%), and remaining comparable to PMF–FFT–SVD (97.2%) and conventional PMF–FFT (98.8%). Overall, these results demonstrate that coherence-optimized sensing provides robust acquisition across a wide Doppler-offset range, which is essential for high-dynamic receivers with large Doppler uncertainty.

Figure 8a shows acquisition probability versus Doppler rate for multiple schemes. The proposed SAGD–FCS remains highly stable across the entire rate range, decreasing only from 97.5% at ${\dot{f}}_{d}=0$ to 96.5% for ${\dot{f}}_{d}\geq 50$ Hz/s. In contrast, Elad-optimized CS and Gaussian-random CS saturate at lower levels (≈89% and ≈64%, respectively), while CFC stays around 71.6–77.6%. PMF–FFT–SVD achieves 94.5% across all rates. SVD-CS maintains high performance but exhibits a mild decline at the maximum tested rate (from 100% at ${\dot{f}}_{d}=0$ to 97.0% at ${\dot{f}}_{d}=1000$ Hz/s). Figure 8b summarizes performance at ${\dot{f}}_{d}=1000$ Hz/s: SAGD achieves 96.5%, outperforming Elad (89.0%), Gaussian random (64.0%), and CFC (75.6%), and remaining comparable to SVD-CS (97.0%) and PMF–FFT–SVD (94.5%). Overall, this experiment confirms robustness against Doppler-rate variations, validating the receiver’s ability to handle signals that deviate from the constant-Doppler assumption.

### 5.6. Positioning vs. Literature Baselines

To position SAGD–FCS within the broader compressed-acquisition literature, we compare it against representative state-of-the-art schemes that either target high dynamics via preprocessing (PMF–FFT–SVD) or reduce the search burden via compressed acquisition (CFC and SVD-CS), under the same Doppler dictionary and coherent-integration setting.

Figure 9 reports acquisition probability versus SNR for multiple methods. Several quantitative observations can be made. First, SAGD–FCS consistently outperforms unoptimized CS baselines under weak signals: at SNR=−16 dB, SAGD achieves 84.6%, while Gaussian-random and Elad-optimized CS yield 36.6% and 60.0%, respectively. Second, SAGD–FCS reaches high acquisition reliability in the moderate-SNR regime: it exceeds 95% at SNR=−15 dB (96.6%) and approaches 100% for SNR≥−14 dB. In terms of the 90% acquisition threshold, SAGD–FCS requires SNR≥−15 dB, compared with −14 dB for CFC, −16 dB for SVD-CS, and −18 dB for PMF–FFT–SVD in this configuration. Taken together with the runtime evidence in Table 2 and the high-dynamic robustness results in Figure 7 and Figure 8, these results demonstrate a superior accuracy–complexity trade-off. By explicitly shaping the sensing operator’s coherence structure offline, SAGD–FCS offloads computational burdens, ensuring competitive acquisition performance with a significantly more lightweight online implementation.

### 5.7. Impact of Compression Ratio and Sparsity Level

Finally, we comprehensively evaluate the impact of measurement length *M* (compression ratio) and assumed sparsity level $K_{\mathrm{sparse}}$ on acquisition robustness under various Doppler uncertainties, thereby providing practical guidelines for parameter selection in real-world high-dynamic GNSS receivers.

Figure 10 studies the impact of the compression ratio on acquisition performance for $N_{f}=256$, with *M* swept from 64 to 224 (compression ratio 25–87.5%). For the SAGD-optimized sensing matrix, the SNR required to achieve 90% acquisition exhibits a clear diminishing-returns trend as *M* increases: approximately −11dB at M=64, −14dB at M=128, and −16dB at M=224. Considering both detection performance and online runtime reported in Table 2, we recommend using M≥192 in weak-signal conditions (e.g., SNR<−15dB), while M∈[96,128] offers a favorable performance–complexity trade-off for moderate-to-high SNR.

Figure 11 evaluates the sensitivity to the assumed sparsity level under $N_{f}=256$ and M=192, where $K_{\mathrm{sparse}}$ is swept from 1 to 10 to account for possible multipath components and residual interference beyond the single-dominant-path setting. The SAGD-optimized matrix remains stable across all tested $K_{\mathrm{sparse}}$: at SNR=−17dB, the acquisition probability stays within 62.8–71.0%, and at SNR=−15dB it remains in the 93–97% range. Moreover, the SNR required to reach 99% acquisition is consistently around −14dB for all $K_{\mathrm{sparse}}$, indicating negligible sensitivity to sparsity variations.

In contrast, the Gaussian matrix attains only 20.27% average detection probability at SNR=−17dB (averaged over $K_{\mathrm{sparse}}$), whereas SAGD reaches 65.30%, corresponding to an improvement of approximately 45 percentage points. These results are consistent with the coherence-driven recovery intuition: reduced mutual coherence improves peak separability and suppresses spurious-peak dominance in the acquisition decision.

## 6. Conclusions

This paper has presented a frequency-domain compressed-sensing acquisition scheme for high-dynamic GNSS receivers. By exploiting the sparsity induced by Doppler uncertainty in a discretized frequency dictionary and employing a carefully optimized low-coherence measurement matrix, the proposed method achieves substantial reductions in computational complexity relative to conventional parallel code-phase search while maintaining detection probabilities in excess of 90% at SNR=−16dB over Doppler offsets up to ±120 kHz. The central contribution is a measurement-matrix design framework based on projected gradient descent with a two-stage annealing schedule, which substantially lowers the maximum coherence. This low-coherence structure enables reliable sparse support recovery using lightweight OMP, avoiding the need for computationally intensive convex optimization. Comprehensive theoretical analysis and Monte Carlo simulations jointly demonstrate enhanced sensitivity and robustness across a broad range of signal lengths, compression ratios, sparsity levels, and Doppler conditions. These properties render the proposed acquisition scheme particularly suitable for implementation in resource-constrained, software-defined GNSS receivers operating in demanding high-dynamic environments.

This study assumes a single-path signal model with additive white Gaussian noise and evaluates Doppler offsets together with a first-order (linear) Doppler-rate variation. The performance under multipath propagation and fading, as well as under more general and faster time-varying Doppler dynamics beyond the linear model, is not fully characterized. These effects will be investigated in future work using more realistic channel models and real IF data.

## Figures and Tables

**Table 1:** Configuration of the test bed.

Hardware	Parameters
CPU	AMD Ryzen 9 9950X CPU @ 4.3 GHz
RAM	KINGBANK DDR5 6400MHz 64GB
Hard Disk	PREDATOR SSD 4T
Graphics Card	NVIDIA RTX 5080

**Table 2:** Online CPU time of different acquisition algorithms.

Acquisition Algorithm	CPU Time (s)
Sliding correlation acquisition	1.379280
Gaussian-random CS acquisition (Gaussian Random)	0.418462
Elad-optimized CS acquisition (Elad Optimized)	0.498942
SVD-optimized acquisition (SVD Optimized)	0.489031
Code–frequency compression acquisition (CFC)	0.137015
CS–SVD–PMF–FFT acquisition (PMF–FFT–SVD)	0.808612
Gaussian-random FCS acquisition (Gaussian Random FCS)	0.095860
Elad-optimized FCS acquisition (Elad Optimized FCS)	0.093853
Proposed SAGD-optimized FCS acquisition (SAGD Optimized)	0.077227

## Data Availability

The original contributions presented in this study are included in the article.
